# A Deep-Learning-Based Scoring Framework for Large-Scale Multi-donor Cardiotoxicity Screening

**DOI:** 10.34133/csbj.0217

**Published:** 2026-09-07

**Authors:** Danny Vu, Andrew Kowalczewski, Sarah D. Burnett, Courtney Sakolish, Xiyuan Liu, Huaxiao Yang, Ivan Rusyn, Zhen Ma

**Affiliations:** ^1^ Department of Biomedical & Chemical Engineering, Syracuse University, Syracuse, NY, USA.; ^2^ BioInspired Institute for Materials and Living Systems, Syracuse University, Syracuse, NY, USA.; ^3^ Department of Veterinary Physiology and Pharmacology, Texas A&M University, College Station, TX, USA.; ^4^ Department of Mechanical & Aerospace Engineering, Syracuse University, Syracuse, NY, USA.; ^5^ Department of Biomedical Engineering, University of North Texas, Denton, TX, USA.

## Abstract

Cardiotoxicity remains a major cause of drug attrition and postmarket withdrawal, yet the vast majority of environmental chemicals to which humans may be exposed remain uncharacterized for cardiotoxicity risk. Human induced pluripotent stem cell (hiPSC)-based testing has been proposed to address this gap. Here, we present an unsupervised deep learning framework for multi-donor cardiotoxicity screening using high-throughput calcium transient recordings from hiPSC-derived cardiomyocytes (hiPSC-CMs). We analyzed data from a library of 1,029 compounds tested in hiPSC-CMs from 5 donors across a concentration range. An autoencoder trained exclusively on baseline signals quantified chemically induced functional perturbations through reconstruction error, bypassing the need for labeled training data while capturing the full spectrum of calcium-handling disruptions. Aggregation of donor-specific scores revealed substantial inter-individual variability in potential cardiotoxicity, underscoring the value of this approach for multi-donor risk prediction. We identified microbiocides, dyes, and pesticides as chemical classes of potential concern, characterized by high toxicity scores and low interdonor variability. This framework establishes a scalable, human-relevant, and genetically diverse platform for cardiotoxicity surveillance across both pharmacological and environmental chemical spaces.

## Introduction

Cardiotoxicity remains a major cause of late-stage clinical failure and postmarket drug withdrawal, accounting for approximately 14% of drugs removed due to adverse cardiac effects [1]. While pharmaceutical compounds have been the primary focus of safety evaluation, the challenge extends to environmental chemicals, which frequently undergo limited or insufficient cardiac safety evaluations but are known to exacerbate cardiovascular disease [2]. Over the past 7 decades, industrial chemical production has increased more than 20-fold, introducing thousands of synthetic compounds into the environment [3], yet the majority of these chemicals remain inadequately characterized for cardiac risk [4]. This disconnect underscores a critical unmet need for scalable, human-relevant experimental models capable of assessing cardiotoxic risk across both pharmacological and environmental chemical spaces while accounting for inter-individual variability [5].

Human induced pluripotent stem cell-derived cardiomyocytes (hiPSC-CMs) have emerged as a transformative platform for cardiotoxicity screening, offering scalability, human specificity, and compatibility with high-throughput functional assays [6–8]. Recent advances enable large-scale screening of drug responses from hiPSC-CMs in 96- and 384-well formats, with quantitative extraction of electrophysiological and contractile features [9–11]. These features have been increasingly integrated with machine learning models to enhance the predictive modeling of drug-induced cardiotoxicity [12–16]. However, these approaches typically decompose the entire signal into individual features, rely on predefined metrics, and may fail to capture higher-order temporal dynamics and subtle nonlinear patterns inherent in raw functional signals.

Deep learning approaches directly operate on raw functional data, bypassing manual feature engineering and capturing subtle patterns that may be overlooked in feature extraction. A hybrid convolutional–recurrent architecture was developed to detect arrhythmic irregularities in field potential recordings from hiPSC-CMs exposed to drugs [17]. A supervised convolutional neural network trained on optical voltage traces has achieved robust classification of drug-induced arrhythmic phenotypes and generated continuous, dose-dependent proarrhythmic risk metrics [18]. More recent studies have expanded these frameworks to integrate multimodal datasets, including high-content imaging and processed electrophysiological signals [19,20]. Despite these advances, current approaches remain constrained by limited chemical diversity, reliance on supervised classification paradigms requiring labeled training data, and insufficient representation of inter-individual variability across genetically distinct donors.

**Fig. 1.**
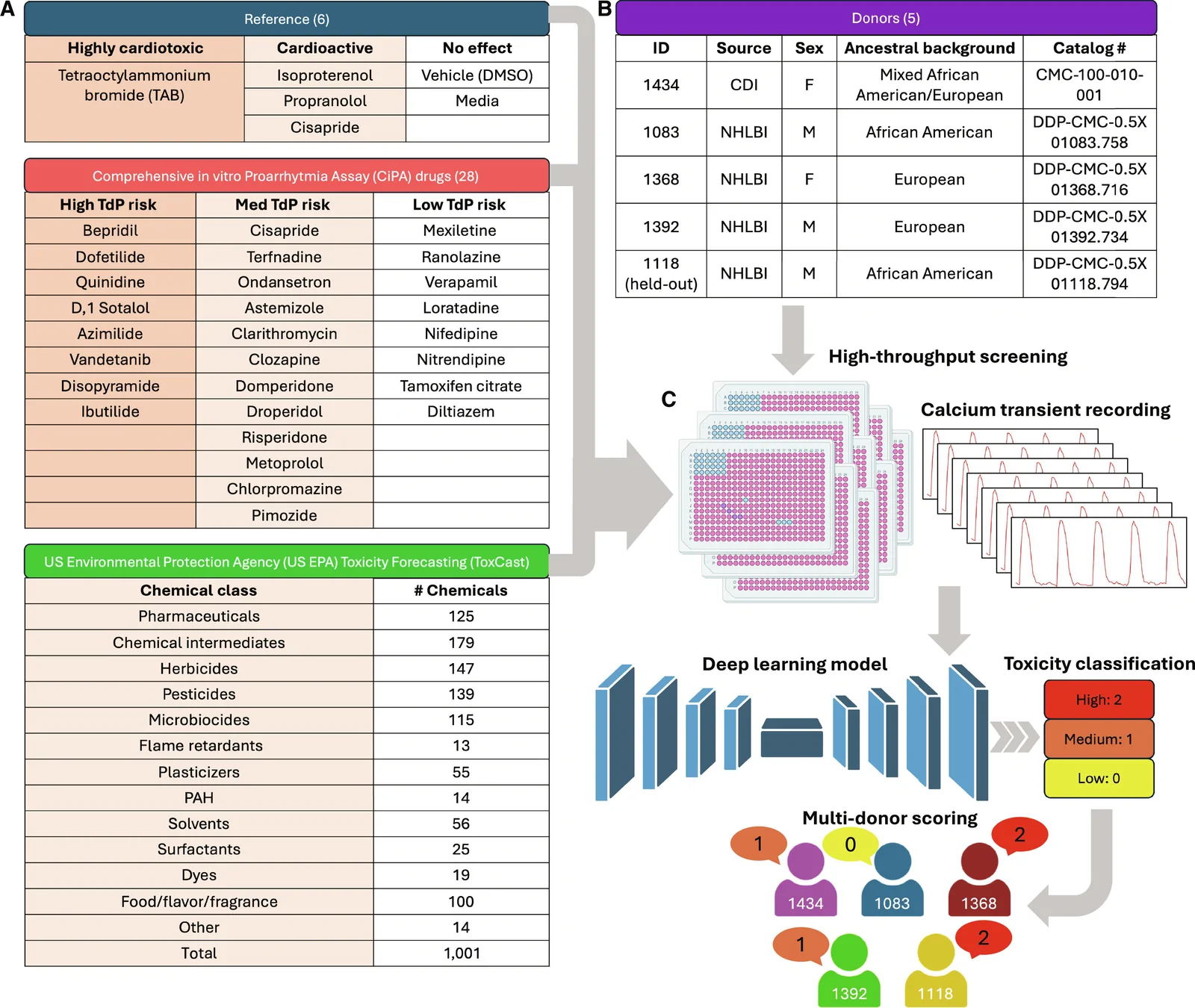
Overview of the multi-donor high-throughput cardiotoxicity screening framework. (A) The chemical library comprised 1,029 test compounds: 28 CiPA drugs and 1,001 ToxCast environmental chemicals across 13 classes. Six reference conditions, four chemical controls, one DMSO vehicle control, and one media-only control, were tested separately for threshold calibration. (B) Compounds were screened in a 384-well format across five hiPSC-CM lines derived from genetically distinct donors. (C) Calcium transient recordings were processed through an autoencoder-based deep learning model, which assigned each signal a reconstruction error-based risk score of 0 (low), 1 (medium), or 2 (high). Scores were aggregated across donors to generate a unified multi-donor cardiotoxicity metric.

Here, we present a multi-donor deep learning framework and apply it to data from a large-scale cardiotoxicity screen using high-throughput calcium transient recordings from hiPSC-CMs derived from 5 human donors. An autoencoder trained exclusively on healthy baseline signals was used to quantify chemically induced functional perturbations through reconstruction error, bypassing the need for labeled training data and enabling sensitivity to a broad spectrum of calcium-handling disruptions. This model was applied to data from a large library of 1,029 compounds that included pharmaceuticals with known cardiotoxicity risk classifications and environmental chemicals from the US Environmental Protection Agency (EPA) Toxicity Forecasting (ToxCast) program representing 13 chemical classes (Fig. 1) [21]. These analyses were performed on the data from each of the 5 donors, and donor-specific reconstruction-error scores were aggregated into a unified multi-donor voting metric to construct multi-donor dose–response relationships. Donor-specific vulnerability analyses revealed substantial inter-individual differences in chemical responses, with microbiocides, dyes, and pesticides emerging as the chemical classes of greatest potential cardiotoxic concern. Together, this framework provides a scalable, human-relevant, and donor-aware strategy for systematic cardiotoxicity risk assessment for both drugs and environmental chemicals.

## Results

### The autoencoder model quantifies chemically induced signal perturbations

The data used in this study were previously published [21]. The screening library comprised 1,029 compounds (Fig. 1A): 28 Comprehensive in vitro Proarrhythmia Assay (CiPA) initiative drugs with defined torsades de pointes (TdP) risk classifications and 1,001 environmental chemicals from the US EPA ToxCast program representing 13 chemical classes. Six reference conditions were tested separately for assay benchmarking and threshold calibration. Compounds were screened in a 384-well format with reference compounds included on each plate to benchmark assay performance and establish cardiotoxicity classification thresholds. Calcium transient recordings were acquired for 100 s at baseline and 90 min post-exposure across 4 concentrations (0.1, 1, 10, and 100 μM) from 5 hiPSC-CM lines derived from genetically distinct donors (Fig. 1B). These recordings were processed through an autoencoder-based deep learning model, which assigned each signal a reconstruction-error-based risk score of 0 (low toxicity), 1 (medium toxicity), or 2 (high toxicity). Scores were then aggregated across donors on a per-compound, per-concentration basis to generate a unified multi-donor cardiotoxicity metric (Fig. 1C). To rigorously evaluate model generalizability, donor 1118 was withheld entirely from model training and reserved for validation, providing a held-out-donor assessment of model generalizability and donor-specific cardiotoxic responses.

**Fig. 2.**
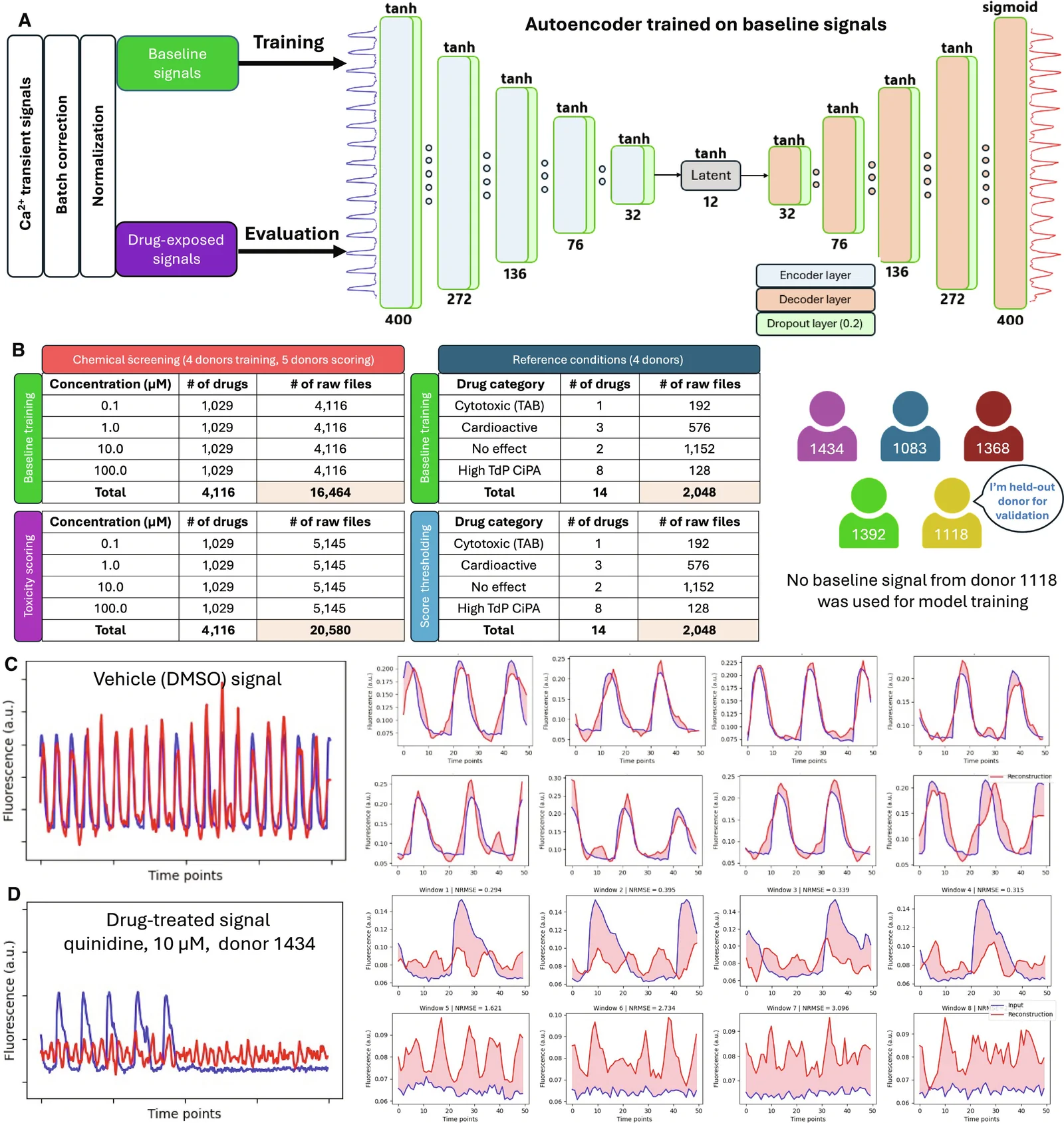
Autoencoder architecture for reconstruction-error quantification. (A) Schematic of the symmetric encoder–decoder architecture comprising 5 fully connected layers in each branch. (B) Dataset design with baseline signals for model training, drug-exposed signals from reference groups for toxicity thresholding, and compound-exposed signals from the chemical screening group for toxicity scoring. Donor 1118 was designated as the held-out validation donor. (C) Representative reconstruction of a vehicle (dimethyl sulfoxide [DMSO])-treated calcium transient signal, showing close agreement between the original (blue) and reconstructed (red) traces across all 8 windowed segments. (D) Representative reconstruction of a drug-treated calcium transient, demonstrating pronounced deviations between original and reconstructed traces, reflecting disrupted calcium-handling dynamics.

To reduce systematic interdonor differences in baseline signal amplitude before model training, ComBat batch correction was applied to the baseline calcium transient recordings used for model training. The mean shape deviation between normalized signals before and after ComBat correction ranged from 0.001 to 0.004, indicating that ComBat reduced interdonor amplitude offsets with minimal effects on waveform morphology (Fig. S1). The corrected baseline signals were used for subsequent autoencoder training and reconstruction-error quantification. The autoencoder-based deep learning framework comprises a symmetric encoder–decoder architecture with 5 fully connected layers in each branch (Fig. 2A). The encoder progressively compressed 400-time-point input signals through hidden layers into a 12-node latent space, while the decoder reconstructed the original signal. The model was trained exclusively on 18,512 baseline calcium transient recordings from 4 donors (1434, 1083, 1368, and 1392), enabling it to learn the intrinsic functional signatures of healthy hiPSC-CMs (Fig. 2B). Loss curves demonstrated rapid initial convergence over 500 epochs, with training loss (mean squared error [MSE] ≈ 0.0048) and validation loss (MSE ≈ 0.0053) remaining closely coupled throughout training, indicating effective generalization without overfitting (Fig. S2a). To further assess model robustness, 5-fold cross-validation was performed on the baseline training set, yielding highly consistent MSE values across all folds, confirming stable reconstruction performance across held-out data partitions (Fig. S2b). The reconstructed signals closely matched the original baseline calcium transient signals (Fig. S2c).

Following training, the autoencoder was applied to calcium transients acquired after chemical exposure. Because the model was trained exclusively on baseline signals, it exhibited limited ability to accurately reconstruct chemically perturbed signals, resulting in pronounced deviations between reconstructed outputs and original inputs. Reconstruction errors were quantified using a windowed normalized root mean square error (NRMSE), which normalizes error relative to the dynamic range of each signal. The autoencoder effectively reconstructed calcium transients from vehicle-treated controls, yielding low NRMSE values (Fig. 2C). In contrast, chemically perturbed signals with pronounced chronotropic effects and altered peak-to-peak intervals produced substantially elevated NRMSE values, reflecting disrupted calcium-handling dynamics (Fig. 2D). Although reconstruction error provided a continuous measure of deviation from baseline calcium dynamics, a structured classification framework was needed to distinguish biologically meaningful chemical effects.

### Multi-donor score aggregation enables dose–response estimation

**Fig. 3.**
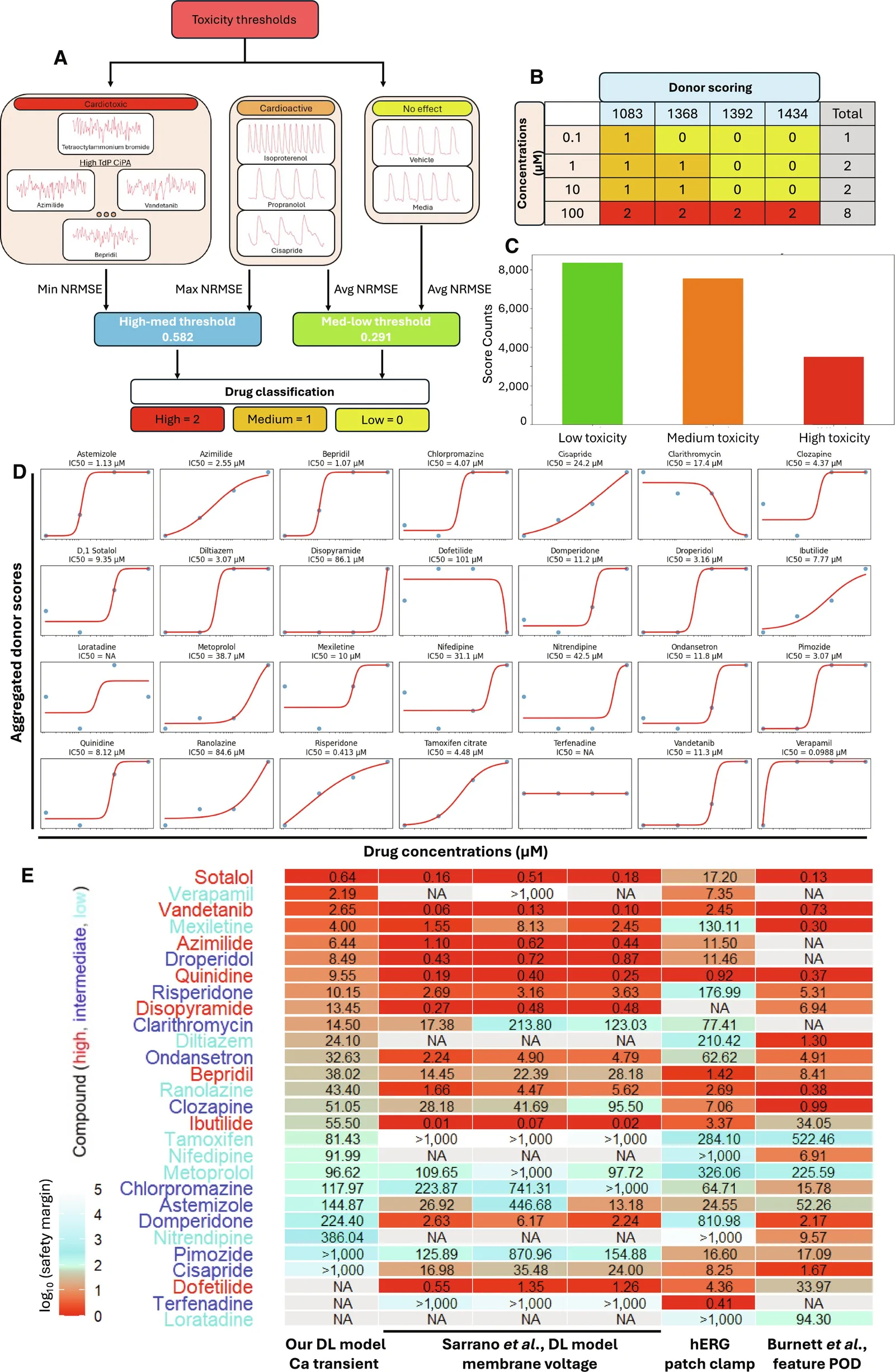
Multi-donor cardiotoxicity scoring for dose–response analysis of Comprehensive in vitro Proarrhythmia Assay (CiPA) compounds. (A) Reference groups used to derive normalized root mean square error (NRMSE)-based cardiotoxicity thresholds for cardiotoxicity classification. (B) Representative donor scoring table illustrating concentration-dependent score aggregation across 4 donors. (C) Distribution of toxicity classifications across all drug-exposed signals. (D) Multi-donor dose–response curves for 25 of 28 CiPA drugs with estimable IC50 values. (E) Safety margin comparison between our deep learning model, Serrano deep learning model, traditional human ether-a-go-go-related gene (hERG)-based approaches, and Burnett feature-based point-of-departure (POD) model.

To stratify drug-exposed signals into cardiotoxicity risk categories, NRMSE-based thresholds were empirically derived from 3 reference groups (Fig. 3A): a high-risk group comprising the cytotoxic compound tetraoctylammonium bromide (TAB, 50 μM) and high-TdP-risk CiPA drugs at 100 μM; a cardioactive group including isoproterenol (10 μM), propranolol (0.5 μM), and cisapride (0.1 μM); and a no-effect group consisting of vehicle and media controls (Fig. S3). The high-medium threshold was set at the midpoint between the lowest NRMSE in the high-risk group and the highest NRMSE in the cardioactive group, yielding a value of 0.582. The medium-low threshold was defined as the midpoint between the mean NRMSE values of the cardioactive and no-effect groups, yielding a value of 0.291. Signals were then assigned scores of 2 (high toxicity), 1 (medium toxicity), or 0 (low toxicity) based on these thresholds, and scores were aggregated across 4 donors per compound per concentration, yielding a maximum possible score of 8 (Fig. 3B). Applying this framework to all chemically exposed signals, the majority were classified as low toxicity (8,357) or medium toxicity (7,561), with a smaller subset categorized as high toxicity (3,486) (Fig. 3C).

Aggregation of donor-specific scores across concentrations enabled construction of multi-donor dose–response relationships. Among the 1,029 compounds tested, 831 exhibited well-defined monotonic dose–response curves suitable for IC50 estimation using the Hill equation. IC50 values were estimable for 25 of the 28 CiPA drugs (Fig. 3D and Table S1). Verapamil exhibited the lowest IC50 (0.1 μM), followed by bepridil (1.07 μM), consistent with their potent disruption of cardiomyocyte electrophysiology. Medium-TdP-risk compounds showed a broader range of IC50 values, from droperidol (3.16 μM) to metoprolol (38.7 μM), reflecting heterogeneous mechanisms of cardioactive perturbation. Low-risk compounds including nifedipine (31 μM) and nitrendipine (42.5 μM) generally exhibited higher IC50 values. Autoencoder-derived IC50 values for CiPA compounds showed consistent trends with reported Ca_V1.2_ patch-clamp potencies for overlapping compounds including bepridil, quinidine, and verapamil, suggesting that the latent features learned by the autoencoder capture perturbations in calcium-dependent electrophysiology (Table S2) [22,23].

To compare IC50 estimates derived from discrete donor scores with those obtained from the continuous reconstruction-error metric, dose–response analysis was also performed using donor-averaged continuous-NRMSE values fitted directly to the Hill equation (Fig. S4a and Table S1). Agreement between continuous-NRMSE and discrete-score IC50 estimates among the CiPA drugs was moderate (Spearman *ρ* = 0.463; Fig. S4b), with notable discrepancies for clarithromycin (discrete: 17.4 μM; continuous: 0.11 μM) and ranolazine (discrete: 84.6 μM; continuous: 0.23 μM). Although continuous-NRMSE fitting provides greater quantitative sensitivity for IC50 estimation, the discrete scoring framework preserves individual donor-level toxicity classifications that are essential for the inter-individual variability analyses central to this multi-donor cardiotoxicity risk framework.

To assess the translational relevance of autoencoder-derived IC50 estimates, safety margins were compared with values from the deep learning model of Serrano *et al.* [18], the feature-based point-of-departure (POD) method of Burnett *et al.* [21], and traditional human ether-a-go-go-related gene (hERG)-based measurements (Fig. 3E) [24]. Compounds with smaller safety margins indicate a narrower therapeutic window, reflecting a higher likelihood of cardiotoxic effects at clinically relevant concentrations. High-TdP-risk compounds demonstrated the strongest overall concordance, with consistently low safety margins across all approaches. Sotalol, azimilide, quinidine, and vandetanib yielded small IC50 values in our autoencoder framework, concordant with their established high-risk classifications and generally low Serrano estimates. In comparison, verapamil showed higher agreement between our model and hERG, while Serrano’s model reported a safety margin >1,000 for one donor, likely reflecting differences in the functional endpoints captured, with calcium transient reconstruction error being inherently sensitive to L-type calcium channel blockade, which is verapamil’s primary mechanism of action [25].

Medium-TdP-risk compounds exhibited the greatest variability across frameworks, consistent with the mechanistic heterogeneity of this category. Ibutilide showed a large discrepancy across frameworks, with the autoencoder yielding the highest safety margin (55.50), compared with substantially lower margins from the Serrano models (0.01 to 0.07) and the hERG-based estimate (3.37). Low-TdP-risk compounds showed better concordance across frameworks, predominantly exhibiting large IC50 values (e.g., nitrendipine and metoprolol) or NA designations (e.g., loratadine) for compounds with minimal functional perturbation at tested concentrations. Overall, concordance between the autoencoder and orthogonal frameworks was strongest for high-TdP-risk compounds, where potent functional disruption produces consistent signals regardless of the assay modality employed. Differences in safety margin estimates across frameworks reflect their distinct mechanistic sensitivities: calcium transient-based reconstruction error captures perturbations in intracellular calcium dynamics, voltage-based endpoints reflect membrane potential changes, and hERG-based margins are limited to potassium channel blockade.

We next plotted safety margins for chemicals in our dataset with available exposure data. Pharmaceuticals were evaluated using population-median *C_max_* values, whereas environmental chemicals were evaluated using median steady-state plasma concentrations derived from ExpoCast exposure estimates and toxicokinetic modeling (Fig. S5). Pharmaceutical compounds exhibited the lowest safety margins of all groups, with median log_10_ values below 2. In contrast, environmental chemical classes exhibited a wide range of safety margins, consistent with the original analysis by Burnett *et al.*

### Donor cardiotoxicity vulnerability across the ToxCast library

**Fig. 4.**
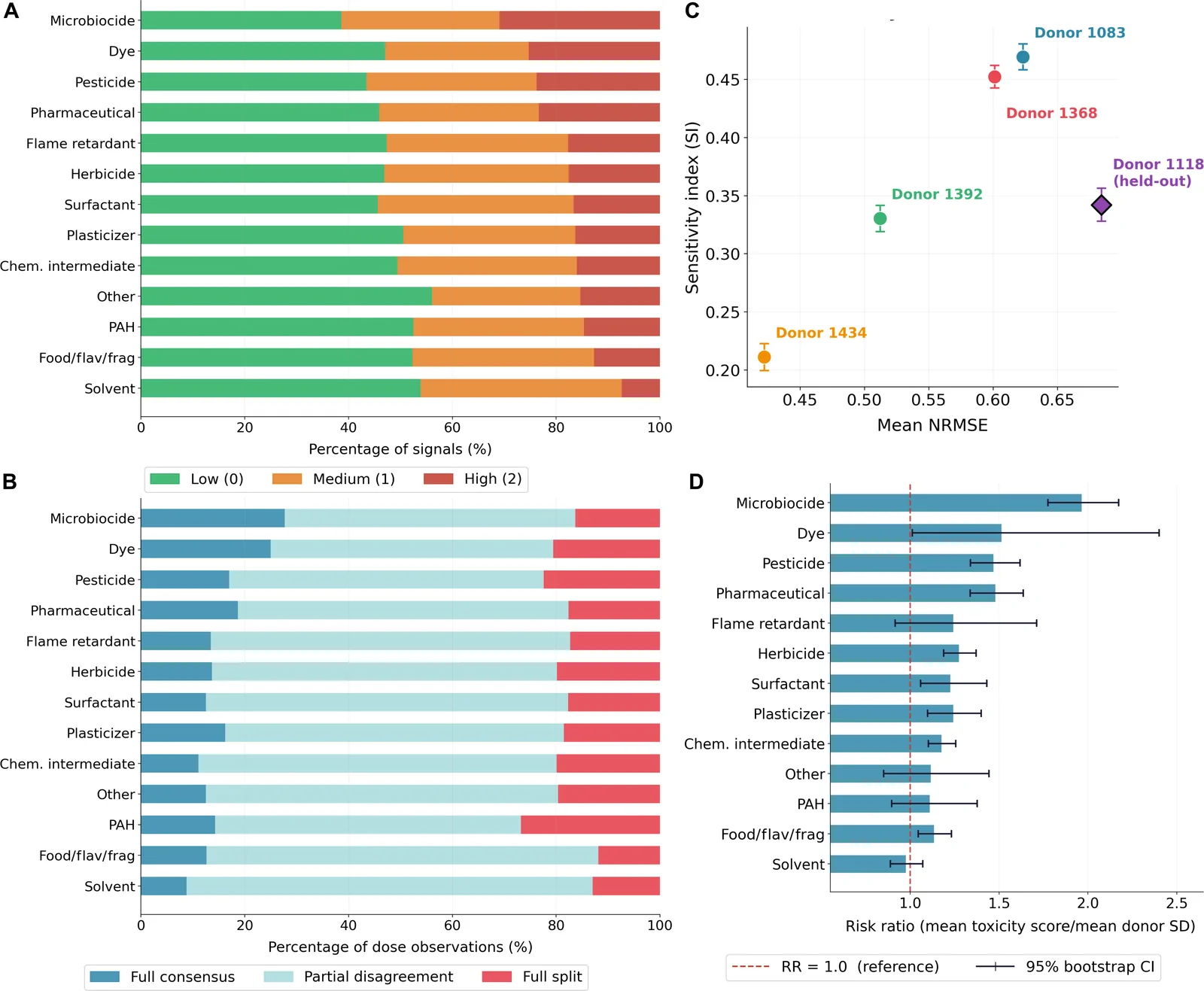
Donor-specific vulnerability and cardiotoxicity profiling for Toxicity Forecasting (ToxCast) chemical classes. (A) Aggregated toxicity score distributions across 13 ToxCast chemical classes, ordered by descending high-toxicity fraction. (B) Donor agreement analysis across ToxCast chemical classes, showing the percentage of dose observations classified as full consensus, partial disagreement, or full split. (C) Scatter plot of sensitivity index (SI) versus mean normalized root mean square error (NRMSE) for each donor. Error bars represent 95% bootstrap confidence intervals (CIs). (D) Risk ratio (mean toxicity score/mean donor standard deviation) for each ToxCast chemical class. Error bars represent 95% bootstrap CIs.

Aggregated toxicity scores across ToxCast chemical classes from all 5 donors, including the held-out donor 1118, revealed distinct class-specific risk profiles and interdonor differences in vulnerability among environmental chemicals (Fig. 4A). Microbiocides exhibited the highest proportion of high-toxicity responses (30.9%), followed by dyes (25.3%) and pesticides (23.8%), while solvents showed the lowest high-toxicity fraction (7.4%) and the highest proportion of low-toxicity responses (53.9%). These patterns are consistent with the intended biological activity of microbiocides and pesticides, which are designed to disrupt cellular function. Class-specific donor responses for representative ToxCast chemicals are shown in Fig. S6. Toxicity score distributions varied substantially across donors, revealing clear differences in cardiomyocyte sensitivity to chemical exposure (Fig. S7). Analysis of donor agreement across chemical classes demonstrated that consensus responses, where all donors agreed on toxicity classification, were most prevalent for solvents (70.4% full consensus) and surfactants (55.2%). Microbiocides showed the lowest full consensus (25.0%) and the highest rate of full split (24.6%) (Fig. 4B), suggesting that individual genetic background substantially influences cardiomyocyte vulnerability to this chemical class.

We calculated the sensitivity index (SI) as a measure of donor vulnerability. First, we conducted a sensitivity test on SI by applying different severity weights (*w* = 1, 2, and 3) to high-toxicity responses for all 5 donors (Table S3). Donor rankings were unchanged with high-toxicity weights of *w* = 2 and 3. Under equal weighting (*w* = 1), donors 1083 and 1368 exchanged the top 2 positions, while donors 1118 and 1392 exchanged the third and fourth positions. Using *w* = 2 for SI calculation, donors 1083 and 1368 exhibited the highest susceptibility, with combined medium- and high-toxicity fractions of 70.9% and 76.8% and vulnerability indices of SI = 0.470 and SI = 0.452, respectively (Fig. 4C). In contrast, donor 1434 displayed the greatest resistance to chemical effects, with 73.2% of signals classified as low toxicity and the lowest vulnerability (SI = 0.211). The held-out donor 1118 exhibited the largest high-toxicity fraction among all donors (29.4%), with an SI of 0.342 and a mean NRMSE of 0.684, positioning it between donors 1392 and 1368 in overall vulnerability. The model successfully captured the elevated cardiotoxic response profile of this unseen donor, whose vulnerability fell within the range spanned by the training donors, demonstrating the robustness and generalizability of the reconstruction-error framework across genetically diverse hiPSC-CM lines.

To identify chemical classes posing the greatest cardiotoxic concern, the risk ratio (RR) of mean toxicity score to mean donor standard deviation (SD) was calculated for each ToxCast category (Fig. 4D). A high ratio indicates that a chemical class produces consistently elevated *in vitro* cardiotoxic responses across the tested donor panel and may therefore warrant higher priority for further hazard characterization. We also calculated 95% bootstrap confidence intervals (CIs) using compound-level resampling to quantify uncertainty in class-level RR estimates. Nine of the 13 chemical classes exhibited RR estimates with 95% CIs entirely above 1.0, indicating cardiotoxic signals that were consistently elevated relative to interdonor variability. Microbiocides showed the highest RR with the narrowest CI, followed by dyes, pharmaceuticals, and pesticides. Notably, solvents were the only class with a 95% CI entirely below 1.0, supporting their comparatively low ranking within this screening framework.

To further resolve compound-level risk profiles within the ToxCast library, individual compounds were plotted on a quadrant map of mean toxicity score versus mean donor SD (Fig. S8). Compounds in the upper right quadrant represented high-risk (high toxicity scores) and high-variability (high donor SD) compounds with heterogeneous susceptibility, which is particularly relevant for environmental risk assessment, where population-level exposure spans genetically diverse individuals. Notable compounds in this category included chlorpyrifos-methyl, mono-(2-ethylhexyl) phthalate (MEHP), simvastatin, 1,2-dimethyl-4-nitrobenzene, fenthion, and myclobutanil, spanning pesticide, plasticizer, and pharmaceutical chemical classes. This finding motivates targeted follow-up studies using expanded donor panels and genetic sequencing to characterize the biological basis of their donor-dependent cardiotoxic responses.

## Discussion

### Unsupervised reconstruction error as a functional cardiotoxicity metric

In this study, we developed a multi-donor deep learning framework for large-scale cardiotoxicity screening, which contrasts with previously reported deep learning approaches that require predefined cardiotoxicity labels for model training [17,18]. By learning the intrinsic functional signatures of healthy cardiomyocytes from baseline recordings alone, the autoencoder generates a continuous, label-free measure of deviation from normal calcium dynamics. Autoencoder-derived IC50 values and safety margins for CiPA compounds showed consistent trends with previous reports based on a deep learning model, a feature-based POD model, and patch-clamp experiments on hERG and Ca_V1.2_ channels. The nonmonotonic dose–response profiles observed for a subset of compounds highlight a broader limitation of IC50 estimation rather than one specific to the present framework. Both the deep learning model of Serrano *et al.* and the feature-based analysis of Burnett *et al.* reported nonestimable (NA) IC50 values for several CiPA compounds, reflecting a broader challenge in modeling complex, nonmonotonic concentration–response relationships for certain drugs rather than a framework-specific limitation. Notably, several compounds classified as NA using our discrete donor scoring approach yielded quantifiable IC50 estimates when continuous-NRMSE values were used for dose–response fitting (Table S4), demonstrating that continuous reconstruction-error analysis can recover concentration-dependent responses that may be obscured by categorical scoring.

### Multi-donor aggregation enables cardiotoxicity risk assessment

Single-donor screening strategies risk conflating donor-specific sensitivity with compound-level cardiotoxicity. By aggregating reconstruction-error-based scores across genetically distinct donors, our framework produces multi-donor dose–response relationships and donor SIs. Donors 1083 and 1368 exhibited the highest vulnerability to cardiotoxicity, while donor 1434 displayed marked resistance. Notably, donor-specific phenotypic differences were evident even before chemical exposure: donor 1083 exhibited a higher baseline beat rate than reference donor 1434, whereas donor 1392 displayed a lower baseline beat rate. Such differences may reflect inter-individual variation in ion-channel expression (e.g., Ca_V1.2_), intracellular calcium-handling machinery (e.g., RyR2), metabolic state, or other intrinsic cellular properties that influence susceptibility to chemical perturbation [26,27]. The successful application of the trained framework to the held-out donor 1118, whose SI fell within the range spanned by the training donors, demonstrates the generalizability of our autoencoder-based donor scoring framework across genetically diverse hiPSC-CM lines. Future integration of transcriptomic profiling, targeted ion-channel characterization, and genetic sequencing of donor lines will help define the molecular determinants underlying the donor-specific vulnerability patterns identified by this framework.

### Differential vulnerability across chemical classes informs environmental risk prioritization

Beyond pharmaceutical cardiotoxicity, our study systematically profiled a large library of environmental chemicals from the US EPA ToxCast program—a chemical space that remains largely uncharacterized for cardiac risk despite widespread human exposure. Among environmental chemical classes, microbiocides, dyes, and pesticides exhibited the highest RRs, exceeded only by pharmaceuticals when all compound categories are considered together. The identification of high-toxicity, high-variability compounds highlights a distinct category of environmental chemicals whose cardiac risk may be concentrated in susceptible individuals. The organophosphate pesticides chlorpyrifos-methyl and fenthion and the plasticizer MEHP are among the most prevalent environmental contaminants in human biomonitoring studies [28,29]. Their high interdonor variability suggests that standard single-donor or population-averaged *in vitro* screening approaches may not adequately capture inter-individual susceptibility differences, highlighting the potential value of multi-donor frameworks for informing the prioritization of compounds for follow-up testing in more comprehensive hazard and risk assessment pipelines.

### Limitations and future directions

Our autoencoder was trained on calcium transient recordings from 4 donors, representing a limited sample of genetic diversity. Expansion to larger donor panels, particularly those enriched for individuals with known cardiac risk factors such as long QT syndrome variants or drug metabolism polymorphisms, would further improve the multi-donor representativeness of the framework. The 8-window segmentation scheme introduces boundary discontinuities at window edges when calcium transient peaks fall near segment transitions, which may locally alter NRMSE at individual window boundaries. While the mean-across-windows aggregation mitigates this effect, overlapping or adaptive windowing strategies that align segment boundaries with beat cycle phases would provide a more physiologically grounded temporal segmentation and represent a technical improvement for future iterations of the framework. Additionally, while calcium transient recordings provide a sensitive readout of intracellular calcium dynamics, they capture a subset of the electrophysiological endpoints relevant to cardiotoxicity assessment. Integration with complementary modalities, such as multielectrode array field potential recordings or contractility measurements, would enable more comprehensive characterization of chemically induced cardiac phenotypes.

The threshold-based classification system, while empirically derived from well-characterized reference compounds, introduces discrete boundaries into what is inherently a continuous biological spectrum; future work could explore probabilistic scoring approaches that more fully leverage the continuous nature of reconstruction-error distributions. Comprehensive mechanistic interpretation of the autoencoder latent representations remains an important challenge and will require integration of explainable artificial intelligence (AI) approaches. Gradient-based attribution methods, such as integrated gradients and SmoothGrad, could identify regions of the calcium transient that most strongly influence latent encoding, linking model sensitivity to specific phases of calcium handling. Similarly, Shapley additive explanations adapted for temporal signals could quantify the contribution of individual signal segments to reconstruction error, while attention-based autoencoders could embed interpretability directly into the model architecture. Incorporating these approaches could reveal whether individual compounds preferentially perturb the systolic rise, peak amplitude, or diastolic decay of calcium transients, thereby extending the framework from cardiotoxicity detection toward the mechanistic interpretation of chemically induced cardiac dysfunction.

## Methods

### Experimental details

Full experimental details of the wet-lab experiments were reported in a previously published study [21]. Briefly, hiPSC-CMs from 5 healthy donors were used to assess inter-individual variability in chemical responses. These 5 donors were drawn from a previously characterized panel of 27 healthy individuals with no known history of cardiovascular disease [27,30]. The hiPSC-CMs from this 27-donor panel exhibit reproducible, donor-specific differences in baseline calcium transient function and drug responses. The 5 donors used here were specifically selected to span the broadest range of baseline cardiomyocyte phenotypes within the panel and thereby maximize representation of inter-individual variability. For instance, relative to the standard donor (1434), donor 1083 exhibited a higher baseline beat rate, while donor 1392 showed a lower beat rate. Cells from each donor were cultured in a 384-well plate format under standardized conditions. Cardiomyocytes were maintained in culture for approximately 14 d prior to experimentation, during which they formed confluent monolayers and exhibited spontaneous, synchronous beating.

Test compounds were administered to hiPSC-CMs using a high-throughput 384-well plate format. Cells were exposed to each compound at 4 concentrations (0.1, 1, 10, and 100 μM) in a final vehicle concentration of 0.5% dimethyl sulfoxide. Compounds were distributed across multiple plates with randomized layouts, and each plate contained an identical set of controls, including vehicle-only and media-only wells. Pharmacological references were incorporated to capture key cardiomyocyte responses. Reference compounds included isoproterenol and propranolol to represent increases and decreases in beat rate, respectively, as well as cisapride to capture delayed repolarization. TAB was included as a cytotoxic control (Fig. S9). These controls were used to define baseline and reference responses for downstream cardiotoxicity classification.

Calcium transient recordings were acquired at baseline and following compound treatment using the FLIPR Tetra Cellular Screening System. Calcium signals were recorded at a sampling rate of 8 frames per second for 100 s, yielding approximately 800 time points per recording. Following baseline acquisition, cells were exposed to test compounds and incubated for 90 min before posttreatment recordings were obtained.

### Data preprocessing

All calcium transient recordings were truncated to the central 400 time points of the original 800 to standardize input length and minimize technical artifacts from the FLIPR Tetra calcium imaging platform, capturing a stable recording window representative of steady-state calcium transient morphology. Recordings were subsequently scaled to the range [0, 1] using min–max normalization to ensure consistent signal amplitudes across recordings and stable model training. To ensure that the model learned physiologically representative calcium transient patterns, recordings with no detectable signal were removed prior to training. A percentile-based amplitude threshold derived from the baseline signal distribution was applied, with the 10th percentile used as the lower cutoff to exclude low-amplitude, noisy recordings. To reduce donor-specific variability in baseline calcium transient signals, batch correction was performed using the ComBat algorithm implemented in the Scanpy package. ComBat applies an empirical Bayes framework to adjust for systematic differences in signal distributions across donors by estimating and removing additive and multiplicative donor-level effects independently at each of the 400 time points. Because ComBat operates on each time point independently without explicitly modeling temporal dependencies, it reduces systematic interdonor differences in signal distributions while retaining the overall waveform morphology of each recording. ComBat was applied exclusively to baseline recordings used for autoencoder training; compound-exposed signals were not batch-corrected, as reconstruction-error quantification relies on deviations from the learned baseline distribution rather than absolute signal amplitude.

### Autoencoder deep learning model

The autoencoder architecture was created and optimized using the KerasTuner package in Python. The final model consisted of a symmetric encoder–decoder structure with 5 fully connected layers in each branch. The encoder progressively compressed 400-dimensional input signals through hidden layers of 272, 136, 76, and 32 nodes into a 12-node latent representation, capturing the most salient features of healthy calcium transient morphology. The decoder mirrored this structure, expanding the latent representation back through layers of 32, 76, 136, and 272 nodes to reconstruct the original 400-dimensional signal. All hidden layers employed hyperbolic tangent (tanh) activation functions to capture nonlinear signal dynamics, and dropout layers with a rate of 0.2 were applied after each dense layer in both the encoder and decoder to reduce overfitting and improve generalization across donors. The output layer used a sigmoid activation function to constrain reconstructed values to the [0, 1] range, consistent with the min–max scaled input data.

The autoencoder was trained using MSE loss to encourage accurate reconstruction of baseline calcium transients. Training was performed for up to 500 epochs with a batch size of 64 and shuffled input data. To prevent overfitting and ensure stable convergence, 2 adaptive training strategies were employed: early stopping restored the best model weights if validation loss failed to improve for 20 consecutive epochs, and a learning rate scheduler reduced the learning rate by a factor of 0.5 after 10 epochs without improvement. Model performance was evaluated using training and validation loss curves to confirm stable convergence without overfitting. To further assess generalizability, 5-fold cross-validation was performed on the baseline training set, with MSE calculated separately for each fold to confirm consistent reconstruction performance across held-out data partitions.

### Reconstruction error

Reconstruction error was quantified using windowed NRMSE, which scales reconstruction error relative to the dynamic range of each individual signal:

$$\text{NRMSE}=\frac{\text{RMSE}}{{\text{signal}}_{\max}-{\text{signal}}_{\min}}$$ (1)

Each recording was divided into 8 consecutive nonoverlapping windows, and NRMSE was calculated independently for each window. The final reconstruction error for each signal was computed as the mean NRMSE across all 8 windows, providing a temporally resolved measure of deviation from learned baseline calcium dynamics.

### Cardiotoxicity threshold

To translate reconstruction-error measurements into biologically interpretable risk categories, NRMSE-based thresholds were empirically derived from reference compounds with well-characterized effects on hiPSC-CM calcium dynamics. Three risk categories were defined: a low-risk category consisting of vehicle-treated or media-control cells; a medium-risk category comprising compounds that markedly alter calcium transient features, specifically isoproterenol (10 μM), propranolol (0.5 μM), and cisapride (0.1 μM); and a high-risk category comprising TAB (50 μM) and high-risk CiPA drugs at the highest tested concentration (100 μM). These compounds were selected to establish benchmark cardiomyocyte responses for comparison with chemically induced effects. Isoproterenol and propranolol represent positive and negative chronotropy, respectively, while cisapride captures changes in repolarization through the decay–rise ratio. TAB serves as a control for cytotoxicity, representing severe disruption of cardiomyocyte function.

Prior to threshold calculation, outlier signals were removed using the interquartile range method to ensure robust estimation of representative reconstruction errors for each reference compound. Signals with NRMSE values exceeding 1.5 times the interquartile range above the third quartile or below the first quartile were excluded. The high-medium threshold was then calculated as the midpoint between the minimum NRMSE of the high-risk category and the maximum NRMSE of the medium-risk category, yielding a value of 0.582. The medium-low threshold was calculated as the midpoint between the mean NRMSE values of the medium-risk and low-risk categories, yielding a value of 0.291. Based on these thresholds, each drug-exposed signal was assigned a toxicity score of 2 (high risk), 1 (medium risk), or 0 (low risk), and scores were aggregated across donors per compound per concentration to yield a maximum possible score of 8.

### Discrete donor score-based IC50 and safety margin estimation

IC50 values were estimated by fitting the multi-donor aggregated dose–response data to the Hill equation using the SciPy optimization package in Python. Safety margins were calculated for CiPA compounds with well-defined dose–response curves as the ratio of the autoencoder-derived IC50 to the clinically relevant maximum plasma concentration (*C_max_*):

$$\text{Safety margin}={\mathrm{IC}}_{50}/C_{\max}$$ (2)

Compounds with smaller safety margins indicate a narrower therapeutic window and a higher likelihood of cardiotoxic effects at clinically relevant concentrations. *C_max_* values were obtained from published clinical pharmacokinetic data [31].

### Continuous-NRMSE-based IC50 estimation

For each compound, NRMSE values were averaged across donors at each concentration and fitted directly to the 4-parameter Hill equation using nonlinear regression. IC50 estimates from the continuous and discrete approaches were compared using Spearman rank correlation of log_10_-transformed IC50 values, with IC50 values capped at 200 μM prior to transformation to limit the influence of poorly constrained fits at the upper concentration boundary. The log_10_ ratio of continuous to discrete IC50 estimates was calculated for all compounds with defined IC50 values under both methods to characterize method-dependent differences.

### Donor vulnerability scoring

To quantify inter-individual differences in cardiomyocyte sensitivity to chemical exposure, an SI was calculated for each donor as a weighted measure of toxicity classifications across all tested compounds:

$$\mathrm{SI}=\frac{w\times N_{\text{high}}+N_{\text{medium}}}{w\times N_{\text{total}}}$$ (3)

where *N_high_*, *N_medium_*, and *N_total_* represent the number of high-toxicity, medium-toxicity, and total scored signals for each donor, respectively, and *w* is a severity weighting parameter applied to high-toxicity responses. Under this formulation, SI values range from 0 (complete resistance, all signals classified as low toxicity) to 1 (maximum vulnerability, all signals classified as high toxicity). To assess the influence of the weighting parameter on donor vulnerability rankings, a sensitivity analysis was performed using values of *w* = 1, 2, and 3. Donor-specific toxicity score distributions were visualized as stacked bar charts showing the percentage of signals classified as low (0), medium (1), and high (2) toxicity for each donor. The relationship between SI and mean NRMSE across donors was visualized as a scatter plot, with each donor represented as a labeled point. The neutral SI threshold (SI = 0.5) was indicated as a reference line to distinguish relatively sensitive from relatively resistant donors.

### Chemical class-level toxicity and donor agreement analysis

To assess cardiotoxic responses across ToxCast chemical classes, toxicity scores were aggregated for all compounds within each chemical category. For each class, the percentage of signals classified as low, medium, and high toxicity was calculated across all donors and concentrations and visualized as a horizontal stacked bar chart ordered by descending high-toxicity fraction [32]. Chemical class annotations were obtained from the US EPA ToxCast database metadata.

Donor agreement was assessed at the per-compound, per-concentration level. For each condition, the distribution of donor toxicity scores was examined and classified into 1 of 3 agreement categories: full consensus, defined as all 5 donors assigned the same toxicity score; partial disagreement, defined as donors split across 2 adjacent toxicity categories; and full split, defined as donors distributed across all 3 toxicity categories or nonadjacent categories. The percentage of dose observations falling into each agreement category was calculated for each chemical class and visualized as a horizontal stacked bar chart ordered by descending full-consensus fraction.

To provide a summary metric for comparative cardiotoxic risk assessment across chemical classes, the RR of the mean toxicity score to the mean donor SD was calculated:

$$\mathrm{RR}=\frac{\text{mean toxicity score}}{\text{mean donor}\mathrm{SD}}$$ (4)

To quantify uncertainty in class-level RR estimates, 95% bootstrap CIs were calculated using compound-level resampling. Within each ToxCast chemical class, compounds were resampled with replacement, with each bootstrap sample containing the same number of compounds as the original class. For each compound, the mean toxicity score was calculated by averaging donor scores (0, 1, or 2) across all 5 donors and 4 concentrations (0.1, 1, 10, and 100 μM). Donor variability was calculated at each concentration as the SD of scores across the 5 donors and then averaged across concentrations. For each bootstrap replicate, the class-level RR was calculated as the mean compound toxicity score divided by the mean compound-level donor SD. This procedure was repeated for 10,000 iterations per chemical class, and the 2.5th and 97.5th percentiles of the bootstrap distribution were used to define the 95% CI. An RR CI entirely above 1.0 was interpreted as evidence that the mean class-level cardiotoxicity score exceeded interdonor variability under this metric. RRs were visualized as a horizontal bar chart with chemical classes ordered by descending ratio value, with error bars representing the 95% bootstrap CIs.

### Compound-level toxicity and variability quadrant analysis

The mean toxicity score, defined as the average of all donor toxicity scores, and the mean donor SD, defined as the average SD of toxicity scores across donors at each concentration, were calculated for each compound across all donors and concentrations. Compounds were plotted in a 2-dimensional scatter plot with mean toxicity score on the *x*-axis and mean donor SD on the *y*-axis. The 75th percentile thresholds for both metrics (mean toxicity score = 0.90; mean donor SD = 0.63) were calculated across all compounds and overlaid as dashed reference lines to define 4 quadrants based on toxicity and variability.

## Data Availability

The code for the deep learning model used to quantify reconstruction error in cardiomyocyte calcium transients is available at https://github.com/Danny-Vu/Syracuse-TAMU. Further information and requests for the complete high-throughput chemical cardiotoxicity screening dataset should be directed to the corresponding author, Zhen Ma (zma112@syr.edu).
