# Supplementary material for: A Deep-Learning-Based Scoring Framework for Large-Scale Multi-donor Cardiotoxicity Screening
Source: Comput Struct Biotechnol J. 2026 Sep 7;35(1):0217. doi: 10.34133/csbj.0217 (PMC13547733; doi:10.34133/csbj.0217)
Supplement: Supplementary file 1 [file csbj.0217.f1.zip › Supplemental Information.pdf]

Supplemental Information

A Deep Learning-Based Multi-Donor Scoring Framework for Large-Scale Cardiotoxicity Screening

Danny Vu[1,2], Andrew Kowalczewski[1,2], Sarah D. Burnett[3], Courtney Sakolish[3], Xiyuan Liu[4], Huaxiao Yang[5], Ivan Rusyn[3], Zhen Ma[1,2]

Affiliations
1 Department of Biomedical & Chemical Engineering, Syracuse University, Syracuse, NY, USA
2 BioInspired Institute for Materials and Living Systems, Syracuse University, Syracuse, NY, USA
3 3 Department of Veterinary Physiology and Pharmacology, Texas A&M University, College Station, TX, USA
4 Department of Mechanical & Aerospace Engineering, Syracuse University, Syracuse, NY, USA
5 Department of Biomedical Engineering, University of North Texas, Denton, TX, USA

Correspondence: Zhen Ma (zma112@syr.edu)
2-171 Center for Science & Technology, Syracuse University, Syracuse, NY, USA 13244

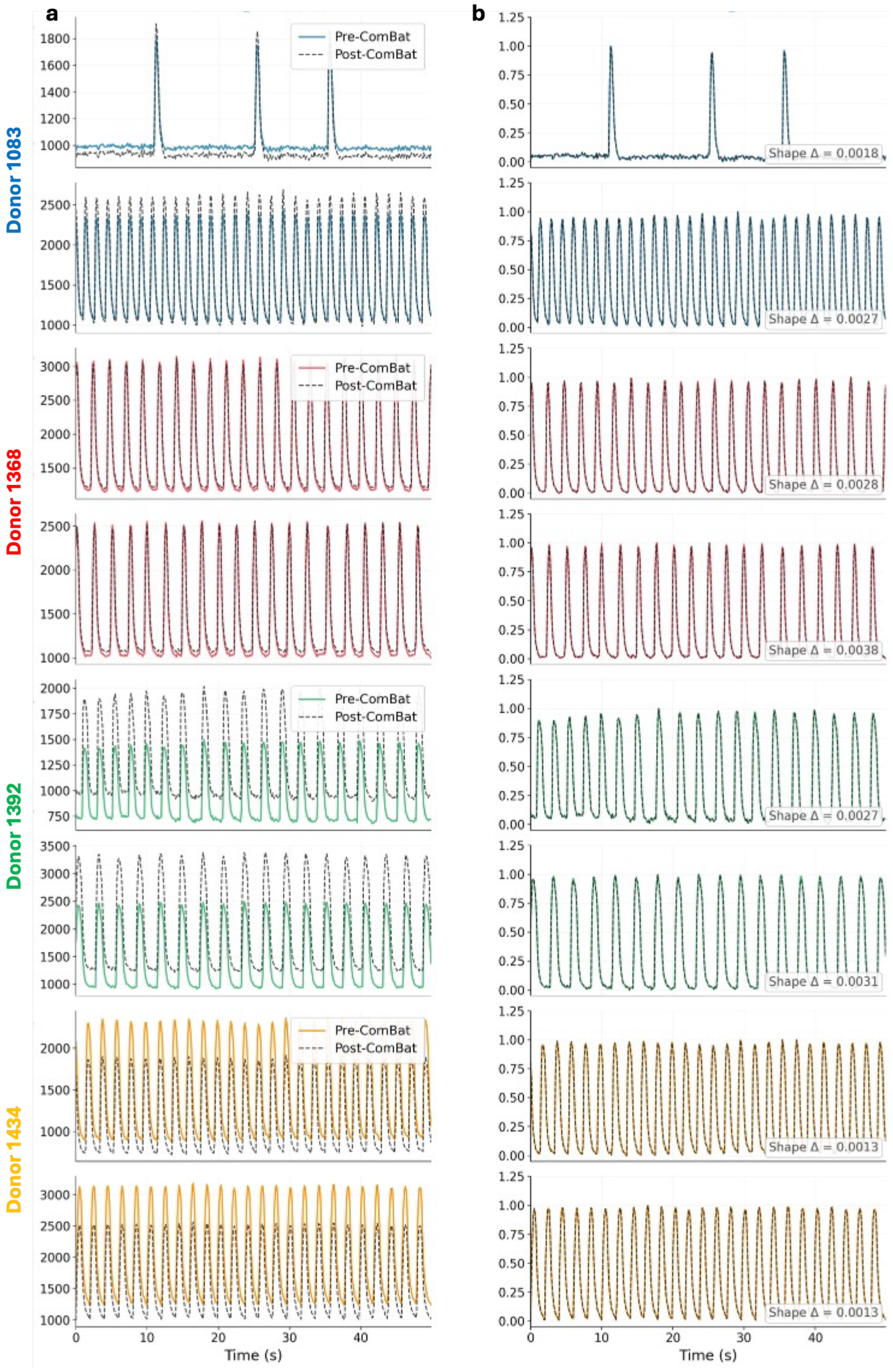

Supplemental Figure 1. Pre- and post-ComBat batch correction of baseline calcium transient signals across four hiPSC-CM donor lines. Representative baseline calcium transient recordings are shown before (colored solid line) and after (black dashed line) ComBat batch correction for two independent traces per donor. (a) Left panels display raw fluorescence signals and (b) right panels display the same signals after min-max normalization.

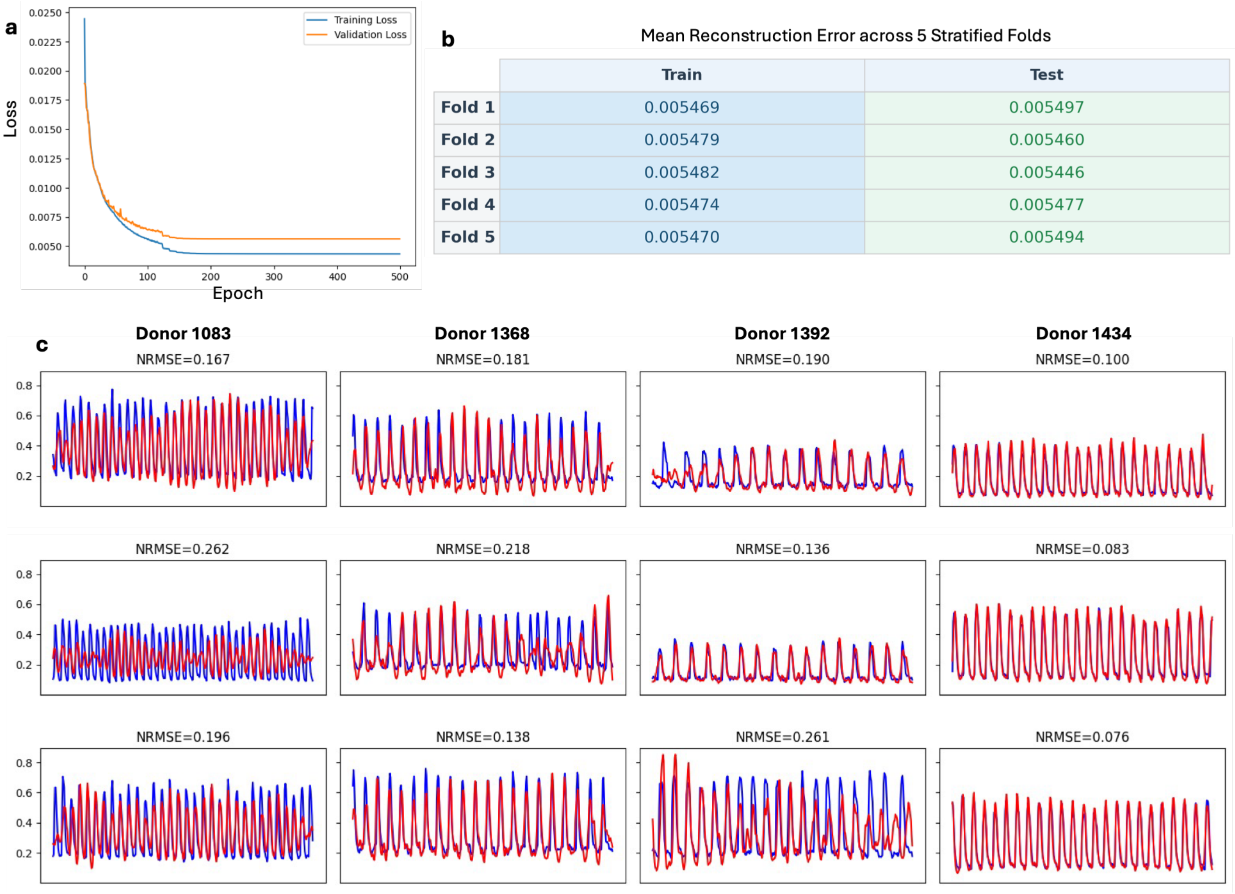

Mean Reconstruction Error across 5 Stratified Folds

	Train	Test
Fold 1	0.005469	0.005497
Fold 2	0.005479	0.005460
Fold 3	0.005482	0.005446
Fold 4	0.005474	0.005477
Fold 5	0.005470	0.005494

Supplemental Figure 2. Autoencoder training performance and cross-validation. (a) Training and validation loss curves over 500 epochs, indicating effective generalization without overfitting. (b) Five-fold cross-validation results showing highly consistent MSE values across all folds (train: 0.00547–0.00548; test: 0.00545–0.00550), confirming stable reconstruction performance across held-out data partitions. (c) Representative reconstructions of baseline calcium transient signals from the validation set, showing close agreement between original (blue) and reconstructed (red) traces.

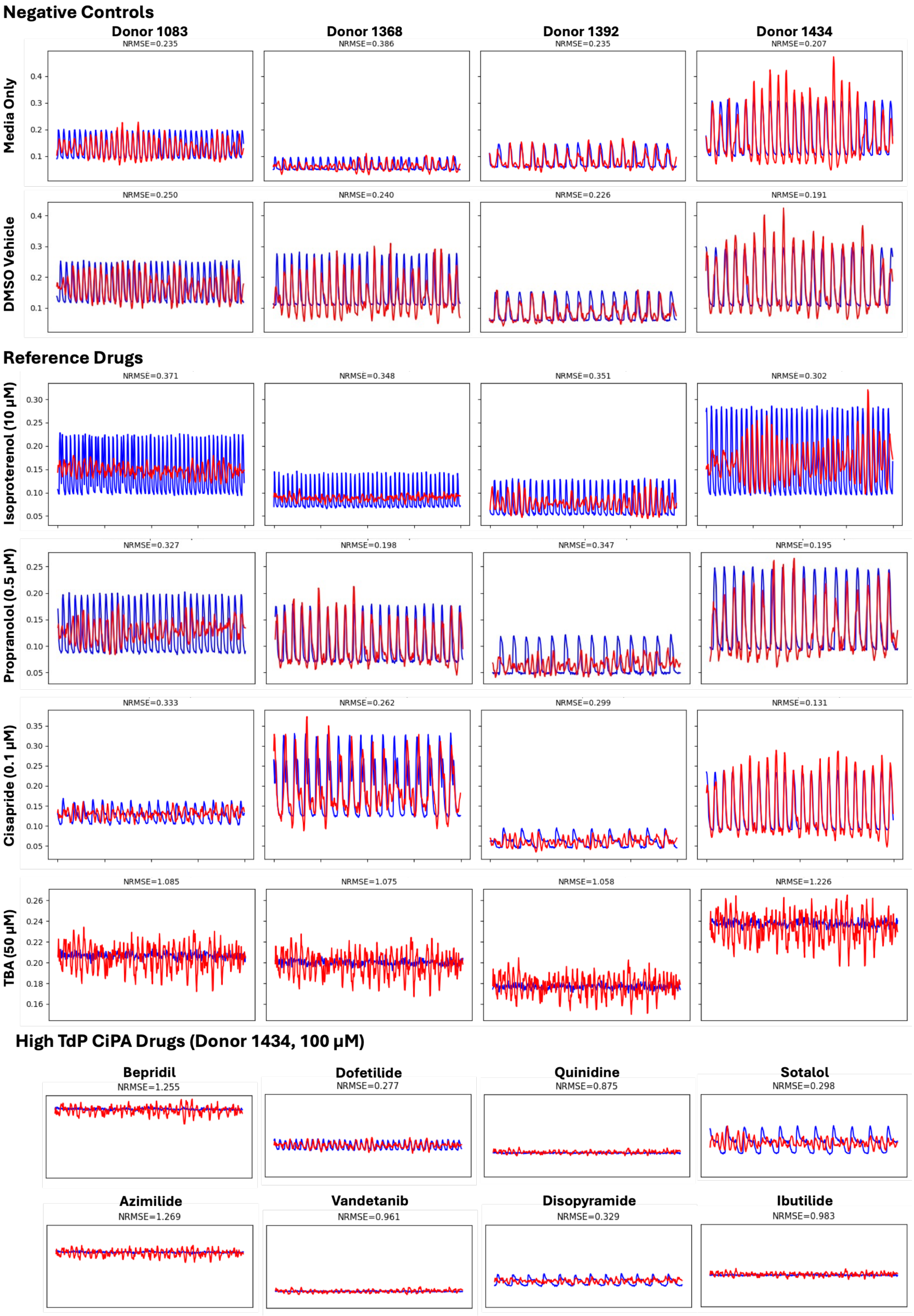

Supplemental Figure 3. Reconstruction error profiles across reference groups. Representative signal reconstruction for negative controls (media only and DMSO vehicle), reference drugs (Isoproterenol, Propranolol, Cisapride and TAB) and high-TdP CiPA drugs at highest concentration (100 µM). The reconstruction errors from these drugs were used to define cardiotoxicity thresholds.

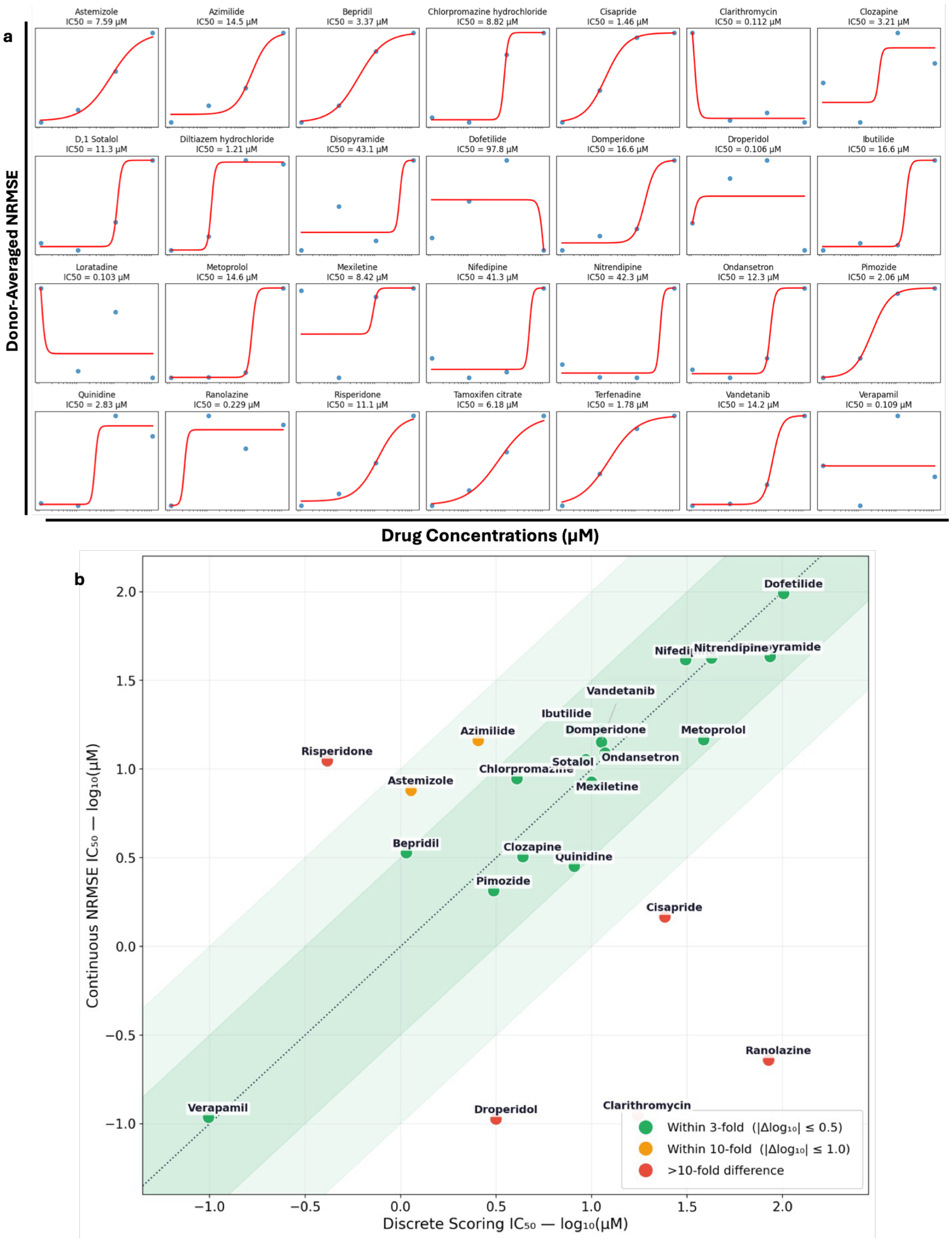

Supplemental Figure 4. Continuous NRMSE-based dose–response analysis. (a) IC50 estimation for CiPA drugs using donor-averaged continuous NRMSE. (b) Correlation of IC50 values between donor-averaged continuous NRMSE method and donor-specific discrete scoring method.

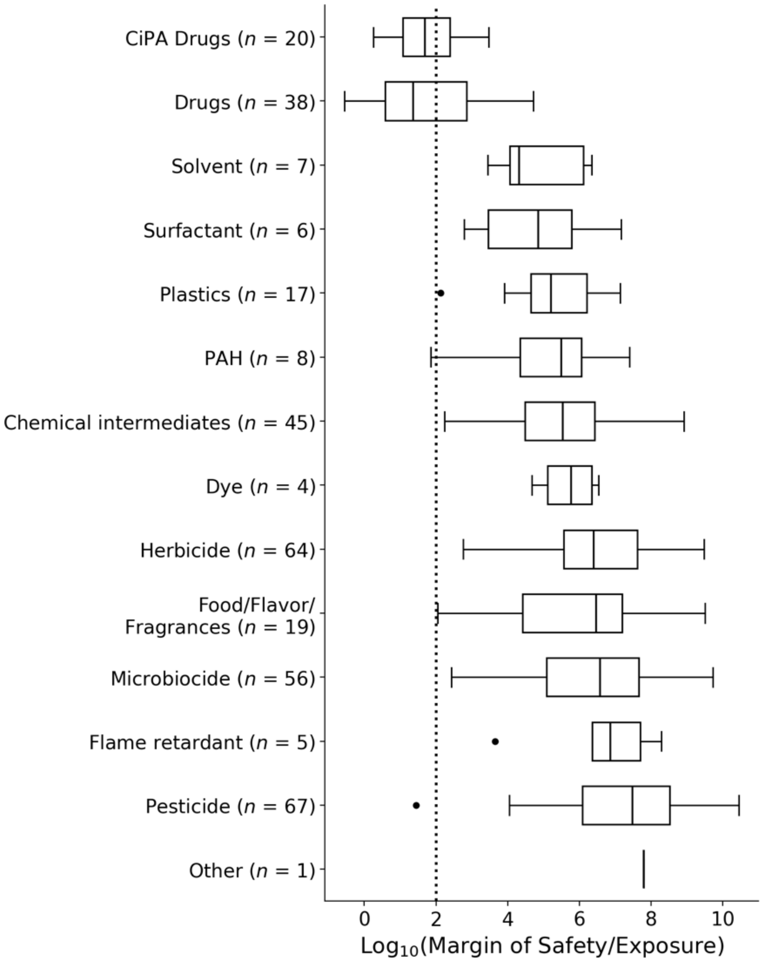

Supplemental Figure 5. Safety margins across chemicals with available exposure data. Pharmaceuticals were evaluated using population median C_{max} values and environmental chemicals were evaluated using the median steady-state plasma concentrations derived from ExpoCast exposure estimates and toxicokinetic modeling

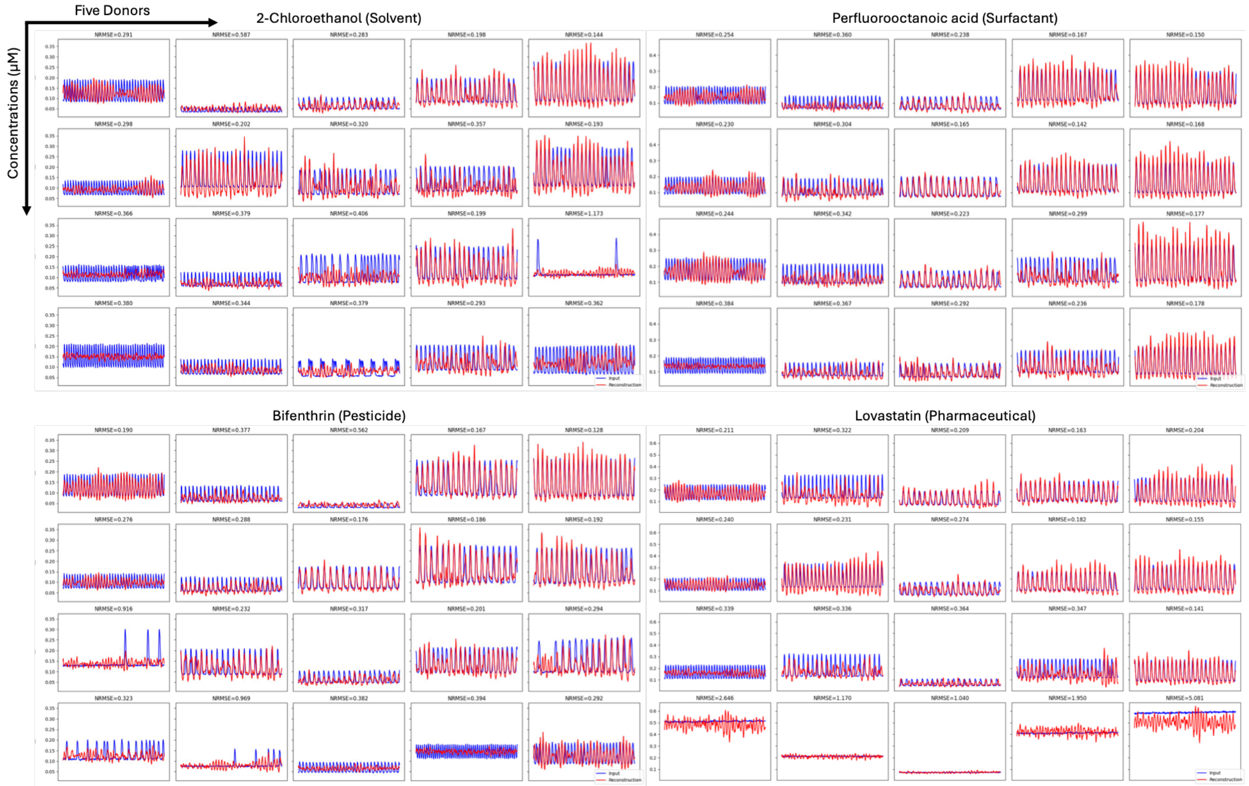

Supplemental Figure 6. Donor-specific drug response differences for representative ToxCast chemicals. Signal reconstruction from five donors, including held-out donor 1118, for four ToxCast chemicals at four concentrations. Inter-donor variability in NRMSE patterns reflects differences in cardiomyocyte sensitivity and calcium handling capacity across genetically distinct hiPSC-CM lines.

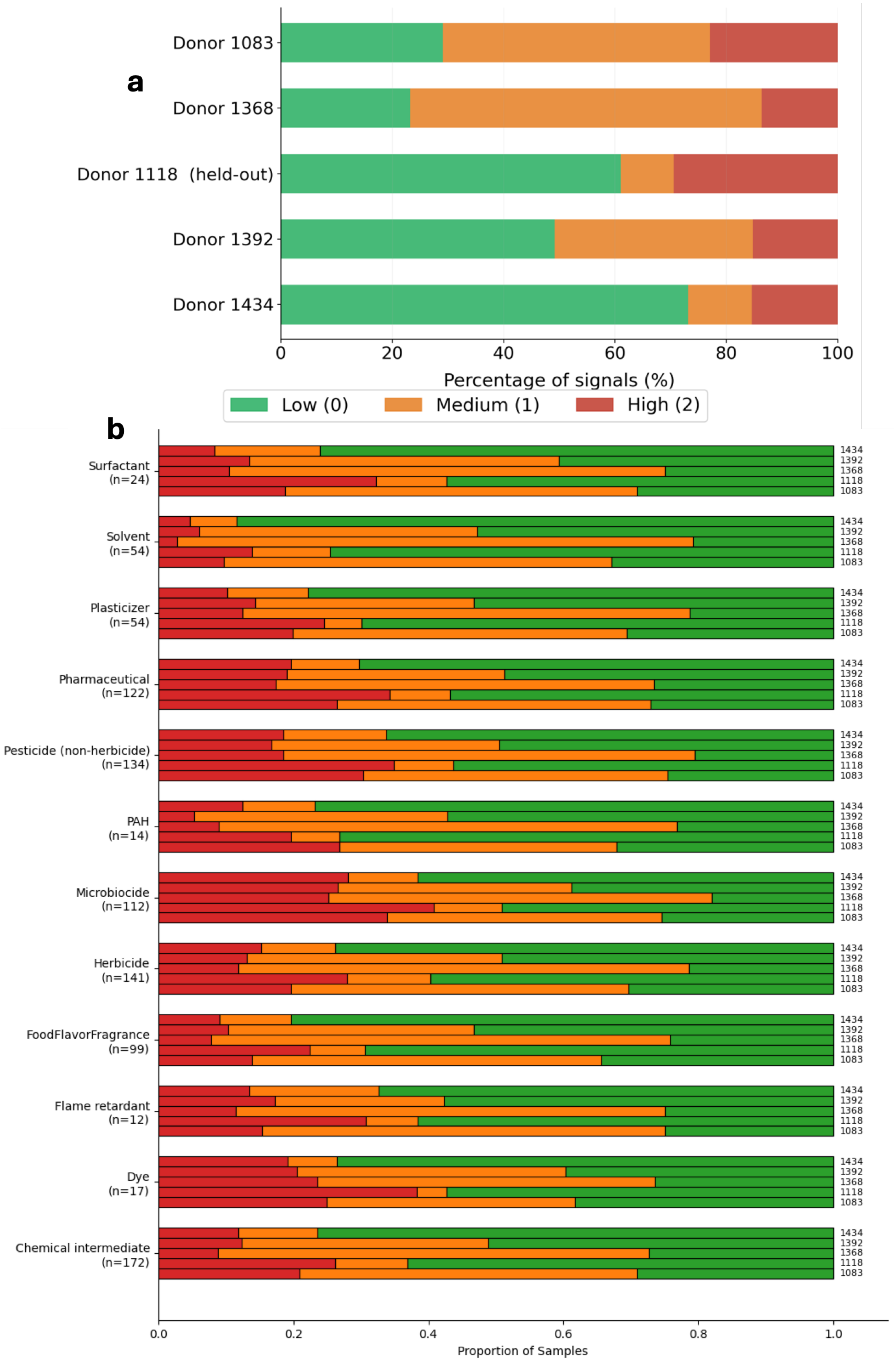

Supplemental Figure 7. Donor-stratified toxicity proportion across ToxCast chemical classes. (a) Donor differences in signal classification into low, medium, and high toxicity, including the held-out donor 1118. (b) Donor-specific signals classification within each ToxCast chemical class.

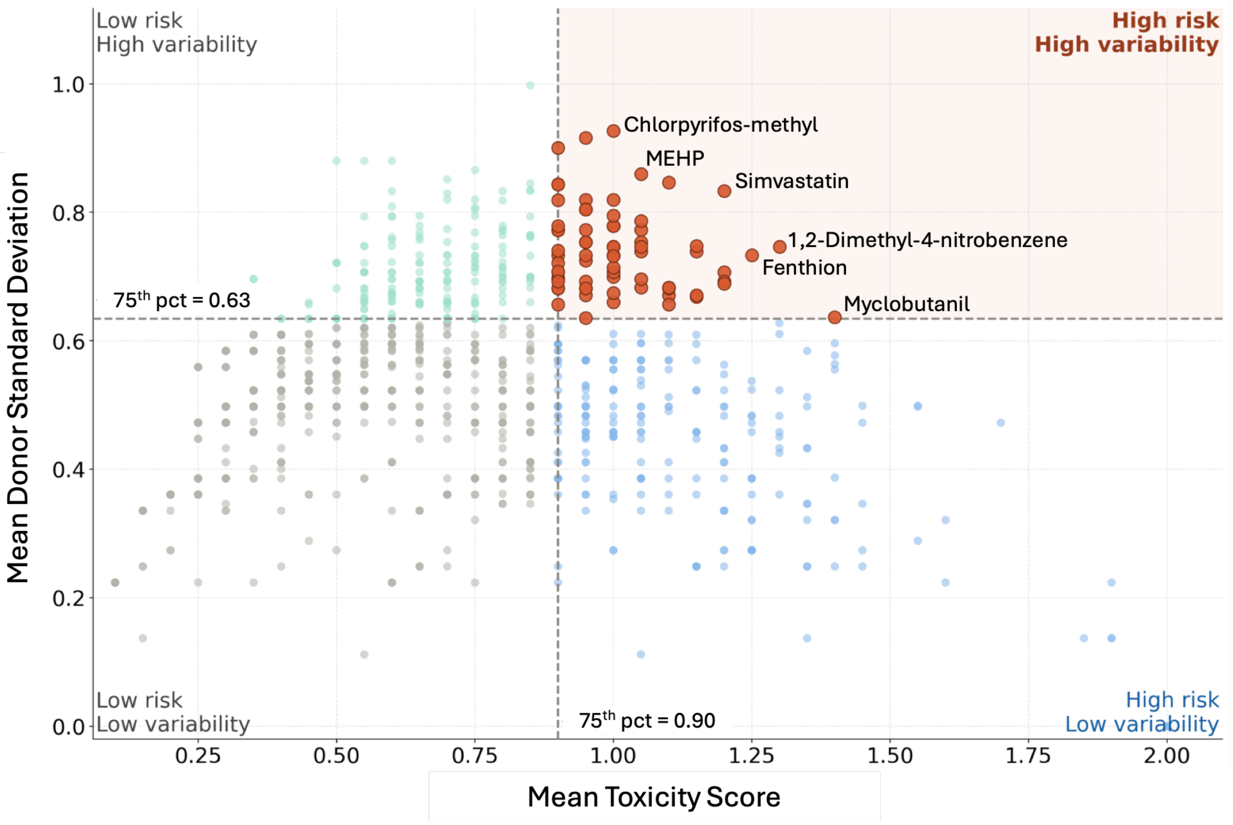

Supplemental Figure 8. Compound-level mean toxicity score versus mean donor standard deviation across the ToxCast library. Dashed reference lines indicate the 75th percentile thresholds for mean toxicity score (0.90) and mean donor STD (0.63), defining four quadrants. Representative chemicals in high risk/high variability quadrant (upper right, shaded) include Chlorpyrifos-methyl (pesticide, banned in European Union), MEHP (plasticizer from food packaging), Simvastatin (prescription drug for high cholesterol), 1,2-Dimethyl-4-nitrobenzene (chemical intermediate in industrial chemical production), Fenthion (pesticide), and Myclobutanil (fungicide).

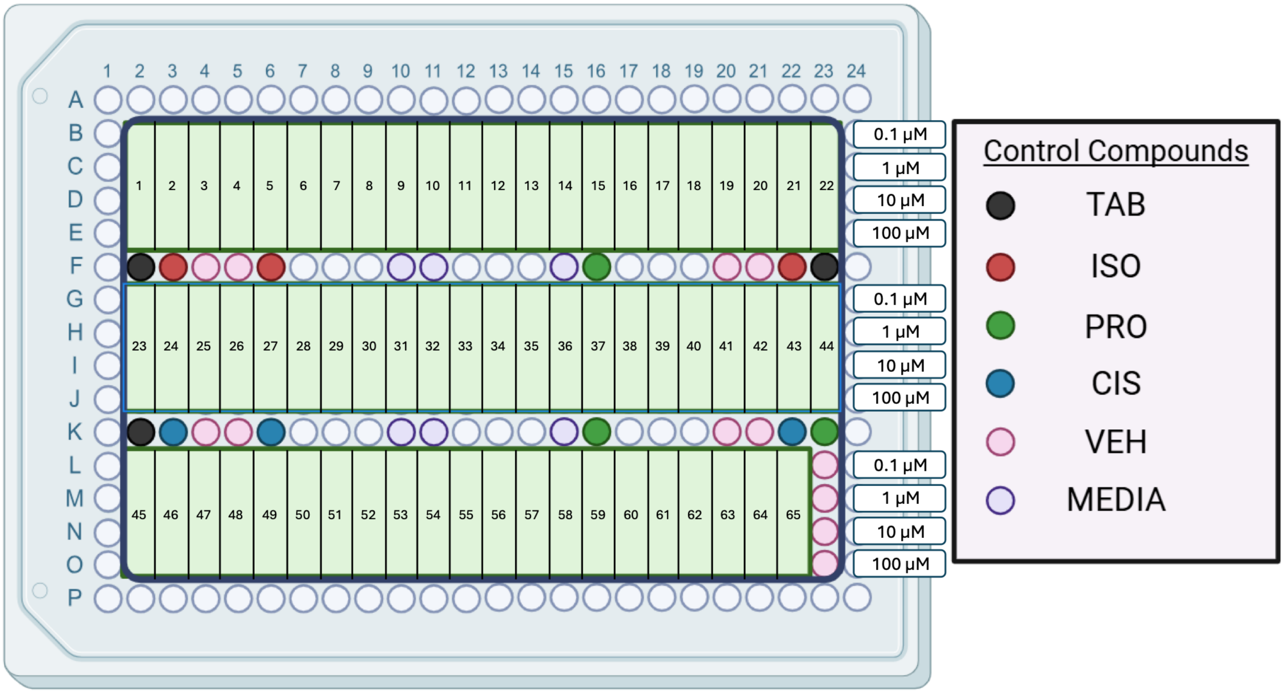

Supplemental Figure 9. Representative Plate Design. One plate in 384 format for high-throughput chemical screening. The plate contains 65 chemical compounds with 4 concentrations, as well as 6 control compounds.

Supplemental Table 1. Safety Margin and IC50 estimates for CiPA drugs using our autoencoder-based deep learning model

	Discrete Score		Continuous NRMSE		
Compounds	IC50 (μM)	Safety Margin	IC50 (μM)	Safety Margin	Plasma Cmax
Bepridil	1.07	33.97	3.37	107.01	0.0315
Dofetilide	NA	NA	NA	NA	0.0021
Quinidine	8.11	9.55	2.83	3.33	0.8492
Sotalol	9.35	0.64	11.29	0.77	14.6864
Azimilide	2.55	6.44	14.49	36.59	0.3960
Vandetanib	11.27	2.65	14.19	3.33	4.2600
Disopyramide	86.06	13.45	43.12	6.74	6.4000
Ibutilide	7.77	55.50	16.56	118.27	0.1400
Terfenadine	NA	NA	1.78	5944.43	0.0003
Ondansetron	11.77	32.63	12.30	34.30	0.3585
Astemizole	1.13	144.87	7.59	972.60	0.0078
Clarithromycin	17.41	14.50	0.11	0.09	1.2000
Clozapine	4.37	51.05	3.21	37.46	0.0856
Cisapride	24.20	9,307.69	1.46	563.40	0.0026
Metoprolol	38.65	96.63	14.64	36.61	0.4000
Chlorpromazine	4.07	117.97	8.82	255.7	0.0345
Pimozide	3.07	1,180.77	2.06	792.66	0.0026
Domperidone	11.22	224.40	16.60	331.92	0.0500
Droperidol	3.16	8.49	0.11	0.29	0.3720
Risperidone	0.41	10.15	11.14	275.76	0.0404
Mexiletine	10.00	4.00	8.42	3.36	2.5032
Ranolazine	84.55	43.40	0.23	0.12	1.9482
Verapamil	0.09	1.97	0.11	2.38	0.0457
Nifedipine	31.10	91.99	41.35	122.33	0.3380
Nitrendipine	42.50	386.04	42.26	384.17	0.1100
Loratadine	NA	NA	0.10	8.34	0.0123

Supplemental Table 2. Comparison of IC50 estimates for overlapped CiPA drugs between our calcium transient-based screening and patch-clamp studies on $Ca_{V1.2}$ ion channel.

Compounds	Autoencoder	$Ca_{V1.2}$ [22]	$Ca_{V1.2}$ [23]
**Bepridil**	1.07	2.8	1.0
**Dofetilide**	NA	NA	26.7
**Quinidine**	8.11	NA	6.4
**Sotalol**	9.35	NA	193.3
**Terfenadine**	NA	0.7	0.9
**Verapamil**	0.09	0.1	0.2

Supplemental Table 3. Sensitivity index under different high-toxicity weighting.

	w = 1		w = 2 ★		w = 3	
Donors	SI	Rank	SI	Rank	SI	Rank
**Donor 1083**	0.710	#2	**0.470**	**#1**	0.389	#1
**Donor 1368**	0.768	#1	**0.452**	**#2**	0.347	#2
**Donor 1118***	0.390	#4	**0.342**	**#3**	0.326	#3
**Donor 1392**	0.508	#3	**0.330**	**#4**	0.271	#4
**Donor 1434**	0.268	#5	**0.211**	**#5**	0.192	#5

Supplementary Table 4 (Separate Excel File). IC50 estimated for all 1029 chemicals using both discrete donor score method and continuous NRMSE method.
